# A cluster analysis of 466 patients demonstrates that glutathione supplementation could preferentially benefit advanced unstable cirrhosis phenotype rather than stable cirrhosis

**DOI:** 10.3389/fphar.2026.1767206

**Published:** 2026-06-30

**Authors:** Cyriac Abby Philips, Aryalakshmi Sreemohan, Ambily Baby, Arif Hussain Theruvath, Tharun Tom Oommen, Rizwan Ahamed, Ajit Tharakan, Philip Augustine

**Affiliations:** 1 Department of Clinical and Translational Hepatology, The Liver Institute, Center of Excellence in Gastrointestinal Sciences, Rajagiri Hospital, Aluva, Kerala, India; 2 Clinical Research Division, The Liver Institute, Center of Excellence in Gastrointestinal Sciences, Rajagiri Hospital, Aluva, Kerala, India; 3 Department of Gastroenterology and Advanced GI Endoscopy, Center of Excellence in Gastrointestinal Sciences, Rajagiri Hospital, Aluva, Kerala, India

**Keywords:** antioxidant, decompensation, hepatitis, portal hypertension, sepsis

## Abstract

**Background:**

Chronic liver disease is characterized by progressive hepatic glutathione depletion and oxidative stress, yet antioxidant therapies have historically shown disappointing clinical efficacy in unselected populations. This therapeutic paradox may reflect inadequate patient stratification and failure to distinguish between acute reversible oxidative injury and chronic persistent inflammation. We hypothesized that glutathione supplementation may preferentially benefit distinct cirrhosis phenotypes with active systemic inflammation.

**Methods:**

We conducted a retrospective cohort study of 466 patients with chronic liver disease who received oral glutathione supplementation (500 mg daily, median 30 days) at a tertiary care center. Primary endpoints were all-cause mortality and change in Model for End-Stage Liver Disease 3.0 (MELD-3) score. We employed conventional statistical methods, survival analysis, multivariable regression, machine learning feature importance, and unsupervised K-means clustering to identify distinct patient phenotypes.

**Results:**

Overall mortality was 10.9% over median 417-day follow-up. At the population level, MELD-3 worsened significantly (mean ΔMELD-3 +2.69, p < 0.001), though 33.5% of patients achieved MELD-3 improvement with low mortality (1.9%). Treatment duration showed no dose-response relationship. Counter-intuitively, patients with higher baseline MELD, bilirubin, INR, and C-reactive protein demonstrated better treatment response. K-means clustering identified four phenotypes, with the “Potential High-Risk Responder” cluster (10.9% of cohort, median MELD-3 27.9) showing highest improvement rate (52.9%) and the only mean MELD-3 improvement (ΔMELD-3–0.85), despite highest mortality (23.5%). An “ideal responder profile” comprising younger patients with alcohol-associated liver disease, elevated baseline MELD, preserved albumin, and elevated inflammatory markers achieved 70.5% improvement rates.

**Conclusion:**

These findings demonstrate that glutathione supplementation may preferentially benefits patients with advanced, acutely unstable cirrhosis characterized by active systemic inflammation rather than stable compensated disease, challenging indiscriminate antioxidant use and supporting a precision-medicine approach targeting inflammation-rich, treatment-responsive phenotypes in chronic liver disease.

## Introduction

Chronic liver disease (CLD) represents a substantial and escalating global health burden, characterized by a progressive decline in hepatic function driven largely by oxidative stress and necroinflammation (Engelmann et al., 2021). Central to this pathogenesis is the depletion of glutathione (GSH), the liver’s predominant intracellular antioxidant, which is critical for neutralizing reactive oxygen species (ROS) generated during metabolic and xenobiotic detoxification (Vairetti et al., 2021). Glutathione is a tripeptide (γ-L-glutamyl-L-cysteinyl-glycine) synthesised intracellularly from glutamate, cysteine and glycine, with cysteine the rate-limiting substrate. Although hepatic GSH pools are maintained predominantly by *de novo* synthesis rather than by direct dietary intake, both preformed glutathione and its sulfur-containing precursors are present in a range of common foods. Cruciferous vegetables (broccoli, Brussels sprouts, cauliflower, kale), allium vegetables (garlic, onions, leeks), spinach, asparagus and avocado contain preformed glutathione, while sulfur-amino-acid-rich foods such as whey protein, eggs, fish and lean poultry provide the cysteine and methionine required for endogenous GSH synthesis. The systemic contribution of dietary glutathione is, however, intrinsically limited: ingested GSH is extensively hydrolysed in the gut lumen and bioavailability of intact GSH after ingestion is modest at best. Glutathione is commercially available in several formulations with substantially different pharmacokinetic profiles. The most widely used is the conventional oral tablet or capsule, typically supplied at 250 mg or 500 mg per unit and prescribed at 250–1,000 mg/day in single or divided doses; this is the formulation used in most clinical studies of glutathione in liver disease to date, including the present one.

Oxidative stress plays a well-established role in the pathogenesis of chronic liver disease, with hepatic glutathione depletion documented across various etiologies including alcohol-associated liver disease, metabolic dysfunction-associated steatotic liver disease, and viral hepatitis. While the theoretical rationale for glutathione supplementation in conditions such as alcohol-associated liver disease (ALD) and metabolic dysfunction-associated steatotic liver disease (MASLD) is robust, given the documented profound depletion of hepatic GSH stores in these states, clinical translation has been historically disappointing (Sacco et al., 2016). Meta-analyses of antioxidant therapies have frequently failed to demonstrate survival benefits, leading to a “therapeutic paradox” where agents with strong biological plausibility show negligible clinical efficacy in unselected populations (Bjelakovic et al., 2010; Singal et al., 2011).

However, this prevailing skepticism may stem from methodological limitations in previous trials, particularly the lack of patient stratification and the failure to distinguish between acute, reversible oxidative injury in liver disease, and chronic, persistent inflammation associated with irreversible fibrosis. Emerging concepts in precision hepatology suggest that “cirrhosis” is not a monolithic entity but comprises distinct phenotypes with varying biological drivers. Specifically, patients with acute decompensation or Acute-on-Chronic Liver Failure (ACLF) exhibit a distinct “storm” of systemic inflammation and oxidative burst that may be uniquely amenable to antioxidant intervention, unlike patients with stable, compensated disease (Mynster et al., 2023; Kronsten and Shawcross, 2025; Trebicka et al., 2019).

While the biochemical rationale for glutathione supplementation is compelling, significant gaps persist in the clinical literature regarding its therapeutic application in cirrhosis. First, despite widespread empirical use of oral glutathione in clinical practice, there is a paucity of large-scale studies examining real-world outcomes in diverse patient populations with chronic liver disease. Second, the optimal duration of glutathione supplementation remains undefined, with treatment courses ranging from weeks to several months in clinical practice without evidence-based guidance. Third, no validated clinical tools exist to predict which patients are most likely to respond to antioxidant therapy, leading to non-selective use across heterogeneous patient populations with varying disease severity and etiologies. Fourth, the relationship between baseline disease severity and treatment response has not been systematically characterized, leaving clinicians without clear patient selection criteria. Finally, the comparative effects of glutathione on different clinical endpoints, including survival, hepatic decompensation events, and biochemical improvement, have not been examined in a competing outcomes framework. These knowledge gaps have resulted in inconsistent clinical practice, potential resource misallocation, and inability to identify patients most likely to derive meaningful benefit from glutathione supplementation.

Our study aimed to bridge these gaps by evaluating the efficacy of glutathione supplementation in a large cohort of patients with diverse chronic liver diseases. The primary aim of this study was to evaluate the clinical outcomes of oral glutathione supplementation in a large real-world cohort of patients with chronic liver disease and to identify predictors of treatment response. Specifically, we sought to: (1) determine whether treatment duration influenced clinical outcomes including mortality, disease severity score trajectory, and hepatic decompensation events; (2) identify baseline clinical and biochemical parameters that predicted favourable response to glutathione supplementation; and (3) characterize distinct patient phenotypes with differential treatment responses.

Unlike prior studies that assessed population-level averages, we utilized machine learning-based cluster analysis to identify specific responder phenotypes and additionally, we sought to define the dose-response relationship to determine if standard treatment durations are sufficient. By delineating the clinical characteristics of the “ideal responder,” our study aimed to challenge the “one-size-fits-all” approach to rationally propose a precision-medicine framework for the use of glutathione in cirrhosis.

## Patients and methods

### Study design and data source

This retrospective cohort study analysed 466 patients with chronic liver disease who received oral glutathione supplementation (500 mg daily for a minimum of 15 days) at a single tertiary care center. Data were extracted from electronic medical records and compiled into a structured database containing 82 clinical variables encompassing demographics, liver disease etiology, baseline laboratory parameters, treatment details, clinical complications, and outcomes. The primary endpoints were all-cause mortality and change in Model for End-Stage Liver Disease 3.0 (MELD-3) score (ΔMELD) from baseline to last follow-up. Secondary endpoints included clinical complications (hepatic encephalopathy, acute kidney injury, infections, and intensive care unit admissions), the Albumin-Bilirubin (ALBI) score, a supplementary measure of hepatic reserve [calculated as (log10 bilirubin [μmol/L] × 0.66) + (albumin [g/L] × −0.085)], and composite outcomes. MELD-3 improvement was defined as any decrease in MELD-3 score from baseline to last follow-up (ΔMELD-3 < 0). The improvement rate was calculated as the proportion of patients achieving MELD-3 improvement within each subgroup.

### Data processing and quality control

Data cleaning procedures included removal of duplicate entries, standardization of categorical variables, and handling of missing values. Missing data were handled using complete-case analysis for primary analyses across variables. Continuous variables were assessed for normality using visual inspection of histograms and the Shapiro-Wilk test. Given the non-normal distribution of most continuous variables, non-parametric methods were employed throughout the analysis. Missing data rates for individual variables are presented in Supplementary Table S1. The primary endpoints had zero missingness. For secondary analyses involving laboratory parameters with missing data, complete-case analysis was employed. Patients with and without available baseline variables were compared on key baseline and outcome variables to assess for systematic differences.

### General statistical methods

Continuous variables were summarized as median with interquartile range (IQR) or mean ± standard deviation (SD) as appropriate. Categorical variables were expressed as frequencies and percentages. Baseline characteristics were compared between groups using the Mann-Whitney U test for continuous variables and the Chi-square test or Fisher’s exact test for categorical variables, as appropriate based on expected cell counts. Within-patient changes in laboratory parameters and clinical scores from baseline to follow-up were assessed using the Wilcoxon signed-rank test for continuous variables and McNemar’s test for paired categorical variables. Effect sizes were calculated as the difference in medians with 95% confidence intervals where applicable. The primary outcome of MELD-3 score change (ΔMELD) was calculated as Follow-up MELD-3 minus Baseline MELD-3, with negative values indicating improvement and positive values indicating worsening.

Associations between continuous variables were assessed using Spearman’s rank correlation coefficient (ρ), given the non-parametric nature of the data. Correlations were specifically examined between drug duration and outcomes (mortality, ΔMELD, improvement status), as well as between changes in inflammatory markers (C-reactive protein, total leukocyte count, neutrophil-to-lymphocyte ratio) and changes in liver function parameters.

Time-to-event analysis was performed using the Kaplan-Meier method. Survival was measured from the date of glutathione initiation to the date of death or last follow-up (censoring). Survival probabilities were estimated at 1-year and 2-year time points. Survival curves were compared between groups using the log-rank test. Subgroup analyses were performed stratifying by baseline MELD-3 category, etiology of liver disease, and MELD-3 trajectory during treatment.

Multivariable Cox proportional hazards regression was performed to identify independent predictors of mortality. The model included clinically relevant covariates selected *a priori*: age, baseline MELD-3 score, baseline albumin, baseline total bilirubin, baseline international normalized ratio (INR), and total duration of drug intake. Results were expressed as hazard ratios (HR) with 95% confidence intervals. Model discrimination was assessed using Harrell’s concordance index (C-index). The proportional hazards assumption was assessed by visual inspection of log-minus-log survival plots.

Univariate and multivariable logistic regression analyses were performed to identify predictors of MELD-3 improvement (defined as ΔMELD-3 < 0) and mortality. Variables with p < 0.1 in univariate analysis or those deemed clinically important were included in multivariable models. Results were expressed as odds ratios (OR) with 95% confidence intervals. Model fit was assessed using the Nagelkerke pseudo-R^2^ statistic.

### Advanced statistical methods

Machine learning feature importance analysis: Random Forest classification models were developed to predict two outcomes: mortality and MELD-3 improvement. Input features included baseline demographics (age), laboratory parameters (MELD-3 score, albumin, total bilirubin, INR, creatinine, sodium, C-reactive protein, haemoglobin, platelet count), treatment duration, and categorical variables (hepatocellular carcinoma status, ascites, prior hepatic encephalopathy, prior infections, and liver disease etiology). Missing values were imputed using median imputation, and features were standardized using z-score normalization. Models were trained using 500 decision trees with a maximum depth of 10 to prevent overfitting. Model performance was evaluated using 5-fold stratified cross-validation with area under the receiver operating characteristic curve (AUC-ROC) as the primary metric. Feature importance was quantified using mean decrease in Gini impurity. Additionally, Gradient Boosting classifiers (200 estimators, maximum depth 5) were trained for comparison.

Cluster analysis for patient phenotyping: Unsupervised K-means clustering was performed to identify distinct patient phenotypes based on baseline clinical characteristics. Input features included age, baseline MELD-3 score, albumin, total bilirubin, INR, creatinine, sodium, C-reactive protein, haemoglobin, and platelet count. Features were standardized prior to clustering. The optimal number of clusters was determined using the elbow method by plotting within-cluster sum of squares against the number of clusters (k = 2–7). Based on clinical interpretability and the elbow plot, k = 4 clusters were selected. Cluster assignments were validated by comparing clinical outcomes (mortality, improvement rate, ΔMELD) across clusters. Cluster phenotypes were characterized by their median baseline parameters and clinical outcomes. Cluster quality was assessed using the silhouette score, Davies-Bouldin index, and Calinski-Harabasz index. The elbow plot (within-cluster sum of squares for k = 2–7) were also presented. Cluster stability was assessed by running k-means with 20 different random seed initializations and computing the Adjusted Rand Index (ARI) across all pairwise cluster assignments.

Subgroup and interaction analyses: Pre-specified subgroup analyses were performed to assess treatment response heterogeneity across clinically relevant patient subgroups. Interaction effects were examined between etiology and baseline MELD-3 severity, age and etiology, hepatocellular carcinoma status and baseline severity, and prior hospitalization status and treatment response. An ‘Ideal Responder Profile’ was defined *post hoc* based on characteristics associated with highest improvement rates.

Composite outcome analyses: Several composite outcomes were defined for clinical relevance: (1) Good outcome: Survival and MELD-3 stable or improved; (2) Poor outcome: Death or severe MELD-3 deterioration; (3) Clinical success: Survival without major complications (no hepatic encephalopathy, acute kidney injury, infections, or ICU admission during follow-up). Composite outcomes were compared across patient clusters and risk score categories.

### Statistical software and significance threshold

All statistical analyses were performed using Python (version 3.12) with the following packages: pandas (version 2.2) for data manipulation, scipy (version 1.14) for statistical tests, statsmodels (version 0.14) for regression analyses, lifelines (version 0.29) for survival analysis, and scikit-learn (version 1.6) for machine learning analyses. Visualization was performed using matplotlib (version 3.9) and seaborn (version 0.13). All p-values were two-tailed, and statistical significance was defined as p < 0.05. No adjustment for multiple comparisons was made given the exploratory nature of the secondary analyses. All confidence intervals were calculated at the 95% level using standard methods.

## Results

### Baseline characteristics

A total of 466 patients with chronic liver disease who received glutathione supplementation (500 mg per day) were included in the analysis. The cohort was predominantly male (85.2%, n = 397) with a median age of 60 years (range: 22–86 years). The most common etiologies of liver disease were metabolic dysfunction-associated steatotic liver disease (MASLD; 51.3%, n = 239) and alcohol-associated liver disease (ALD; 36.5%, n = 170). Hepatocellular carcinoma was present in 24.5% of patients (n = 114). The median baseline MELD-3 score was 16.42, with a mean of 17.53 ± 7.57. Median baseline laboratory values included albumin 3.00 g/dL, total bilirubin 2.70 mg/dL, INR 1.56, creatinine 0.90 mg/dL, and sodium 138 mEq/L. The median duration of glutathione supplementation was 30 days (range: 15–300 days), with a mean of 40.9 ± 36.3 days. Patients were followed for a median of 417.5 days (range: 7–1,533 days), with a mean follow-up of 497.2 ± 359.7 days (Table 1).

**TABLE 1:** Pertinent baseline characteristics of the whole cohort (N = 466).

Characteristic	Value		
Age (years)	59.9 ± 11.0 (range: 22–86)		
Male	397 (85.2%)		
Female	69 (14.8%)		
MASLD	239 (51.3%)		
ALD	170 (36.5%)		
DILI	14 (3.0%)		
AVH	13 (2.8%)		
Other	30 (6.4%)		
HCC present	114 (24.5%)		

Variables	Mean ± SD	Median	Range
Age (years)	59.9 ± 11.0	60.0	22–86
Glutathione duration (days)	40.9 ± 30.4	30.0	15–300
Follow-up duration (days)	497.2 ± 415.9	417.5	7–1,533
Baseline MELD-3	17.5 ± 5.4	16.4	6.4–42.9
Baseline ALBI	−1.50 ± 0.64	−1.48	−3.42-0.07
Baseline albumin (g/dL)	3.09 ± 0.62	3.0	1.6–5.0
Baseline total bilirubin (mg/dL)	4.26 ± 3.97	3.25	0.3–26.1
Baseline INR	1.75 ± 0.59	1.65	0.9–6.2

### Mortality and MELD-3 score changes

**FIGURE 1:**
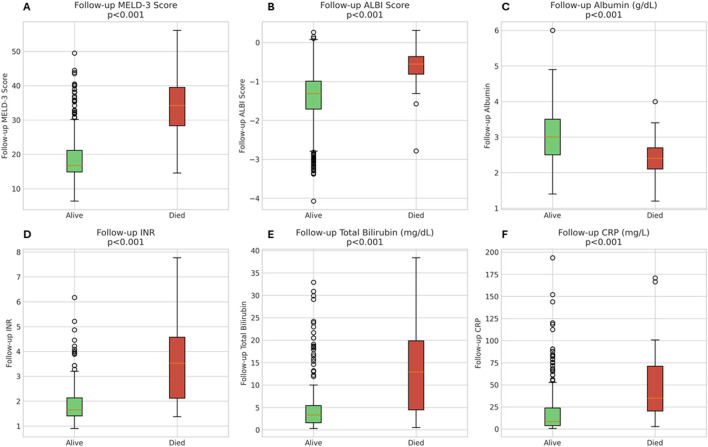
Follow-up clinical parameters stratified by survival status - Box plots comparing follow-up clinical parameters between patients who survived (Alive, n = 415) and those who died (Died, n = 51) during the study period. Parameters shown include **(A)** Follow-up MELD-3 score **(B)** Follow-up ALBI (Albumin-Bilirubin) score **(C)** Follow-up serum albumin (g/dL) **(D)** Follow-up INR (International Normalized Ratio) **(E)** Follow-up total bilirubin (mg/dL), and **(F)** Follow-up C-reactive protein (mg/L). All parameters demonstrated highly significant differences between survival groups (all p < 0.001, Mann-Whitney U test). Patients who died exhibited markedly worse hepatic function at follow-up, with higher MELD-3 scores (median 29.5 vs. 18.0), more negative ALBI scores (median −0.75 vs. −1.35), lower albumin (median 2.40 vs. 2.95 g/dL), higher INR (median 2.90 vs. 1.75), higher bilirubin (median 10.0 vs. 3.5 mg/dL), and elevated inflammatory markers with higher CRP (median 35.0 vs. 15.0 mg/L). Box plots display median (center line), interquartile range (box), 1.5× IQR whiskers, and outliers (circles). These findings demonstrate that follow-up parameters vastly outperformed baseline parameters in predicting mortality, highlighting that disease trajectory during treatment is the dominant factor determining outcomes.

Overall mortality during the follow-up period was 10.9% (n = 51). Kaplan-Meier survival analysis demonstrated 1-year and 2-year survival rates of 91.0% and 86.2%, respectively. The median survival was not reached during the observation period. Despite glutathione supplementation, there was a statistically significant worsening in disease severity at the overall population level. The mean ΔMELD-3 was +2.69 points (median +1.18, p < 0.001 b y Wilcoxon signed-rank test), indicating overall disease progression. Significant deterioration was observed in individual MELD-3 components: total bilirubin increased by a mean of +1.73 mg/dL (p < 0.001), and INR increased by +0.38 (p < 0.001). Serum albumin decreased slightly (−0.09 g/dL, p < 0.001), while creatinine and sodium showed minimal changes (Table 2; Figure 1).

**TABLE 2:** Complications at baseline vs. follow-up.

Complication	Baseline n (%)	Follow-up n (%)	Change
Ascites	139 (29.8%)	186 (39.9%)	+10.1%
Hepatic encephalopathy	45 (9.7%)	98 (21.0%)	+11.3%
Acute kidney injury	40 (8.6%)	75 (16.1%)	+7.5%
Variceal bleeding	71 (15.2%)	63 (13.5%)	−1.7%
Infections/sepsis	46 (9.9%)	121 (26.0%)	+16.1%
ICU admission	34 (7.3%)	148 (31.8%)	+24.5%

**FIGURE 2:**
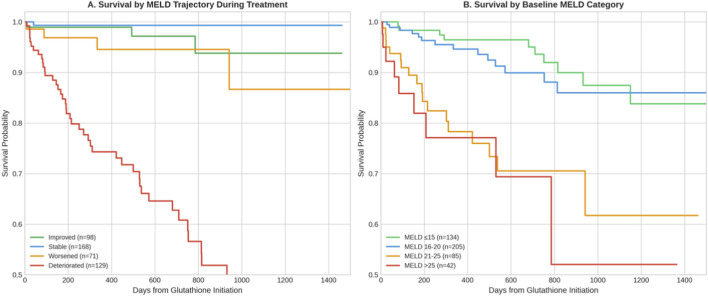
Kaplan-Meier survival analysis by MELD-3 trajectory and baseline severity–Demonstrating **(A)** Survival probability stratified by MELD-3 trajectory during glutathione treatment and **(B)** Survival probability stratified by baseline MELD-3 category. Panel A shows four distinct trajectory groups: Improved (ΔMELD-3 < −2, n = 98, green line) with 1-year survival of 98.9% and overall survival of 96.9%; Stable (ΔMELD-three to two to +2, n = 168, blue line) with 1-year survival of 99.3% and the best overall survival of 99.4%; Worsened (ΔMELD-3 +2 to +5, n = 71, orange line) with 1-year survival of 94.2% and overall survival of 87.3%; and Deteriorated (ΔMELD-3 > +5, n = 129, red line) with markedly reduced 1-year survival of 74.3% and overall survival of 66.7%. Log-rank test: p < 0.001. Panel B demonstrates survival stratified by baseline disease severity: MELD-3 ≤15 (n = 134, green line, 1-year survival 96.4%), MELD-3 16–20 (n = 205, blue line, 1-year survival 94.6%), MELD-3 21–25 (n = 85, orange line, 1-year survival 78.3%), and MELD-3 >25 (n = 42, red line, 1-year survival 77.1%). Log-rank test: p < 0.001. Patients with improved or stable MELD-3 trajectories demonstrated near-perfect survival (>98%), while those with severe deterioration (ΔMELD-3 >5) had only 74% 1-year survival, validating serial MELD-3 monitoring as the primary method for assessing treatment response. Follow-up duration is shown in days from glutathione initiation. These survival curves demonstrate a clear gradient in outcomes based on both baseline severity and on-treatment disease trajectory.

Stratification by MELD-3 trajectory revealed marked heterogeneity in treatment response. Approximately one-third of patients (33.5%, n = 156) achieved MELD-3 improvement (ΔMELD-3 < 0), while 23.6% (n = 110) remained stable (ΔMELD-3 0–2) and 42.9% (n = 200) worsened (ΔMELD-3 > 2). The combined improved/stable group (57.1%) demonstrated excellent survival with only 1.5% mortality, whereas the worsened group accounted for 92% of all deaths with a mortality rate of 23.5%. Further analysis by MELD-3 trajectory severity showed a clear gradient in outcomes: patients who improved (ΔMELD-3 < −2, n = 98) had 3.1% mortality and 98.9% 1-year survival; those who remained stable (ΔMELD-three to two to +2, n = 168) had the lowest mortality at 0.6% and 99.3% 1-year survival; patients who worsened moderately (ΔMELD-3 +2 to +5, n = 71) had 5.6% mortality; and those who deteriorated severely (ΔMELD-3 > +5, n = 129) had 33.3% mortality with only 74.3% 1-year survival (Figure 2). As a sensitivity analysis, a stricter improvement definition of ΔMELD-3 ≤ −2 (at least two-point decrease, representing a clinically meaningful change) was applied. Under this definition, 98 patients (21.0%) achieved strict improvement. The severity-response gradient was preserved and became more pronounced: strict improvement rates were 6.0% for baseline MELD-3 ≤15, 17.5% for 16–20, 40.7% for 21–25, and 47.6% for >25, confirming that the association between higher baseline severity and greater improvement is robust to the choice of improvement threshold.

Acute viral hepatitis (AVH, n = 13, 2.8%) was the only etiologic subgroup with mean MELD-3 improvement (ΔMELD −1.18), which likely reflects spontaneous disease resolution rather than any treatment effect. These patients had underlying chronic liver disease with superimposed acute viral hepatitis as a precipitating event. Sensitivity analysis excluding AVH patients yielded virtually identical results (improvement rate 33.3% vs. 33.5%, mortality 11.3% vs. 10.9%, mean ΔMELD +2.80 vs. +2.69), confirming that their inclusion does not meaningfully influence the findings.

A dedicated HCC subgroup analysis showed that HCC patients (n = 114, 24.5%) had numerically lower MELD-3 improvement rates (28.9% vs. 34.9%, p = 0.29) and greater mean ΔMELD-3 (+3.79 vs. +2.34, p = 0.055) compared to non-HCC patients, with no difference in mortality (10.5% vs. 11.1%, p = 1.00). MELD-3 improvement in HCC patients—including within Cluster two where HCC prevalence was 34.9% — likely reflects tumour-directed therapies or fluctuations in hepatic reserve unrelated to antioxidant supplementation, and should not be interpreted as evidence of glutathione efficacy.

## Glutathione duration and treatment outcomes

**FIGURE 3:**
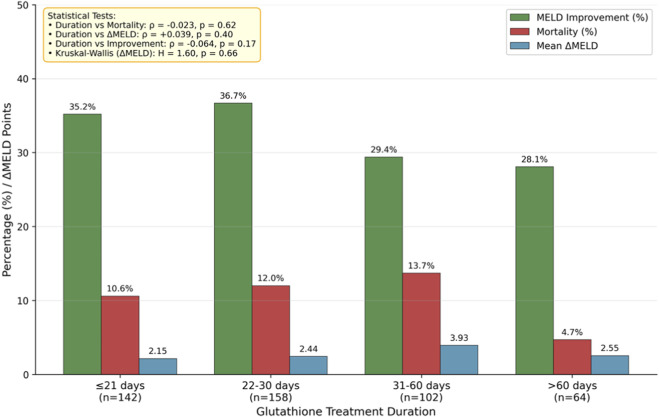
Absence of dose-response relationship between glutathione duration and clinical outcomes–Bar graph demonstrating the lack of association between glutathione supplementation duration and clinical outcomes. Patients were stratified into four duration categories: ≤21 days (n = 142), 22–30 days (n = 158), 31–60 days (n = 102), and >60 days (n = 64). Green bars represent MELD-3 improvement rate (%), red bars represent mortality rate (%), and blue bars represent mean ΔMELD. Statistical tests displayed in the yellow box demonstrate no significant relationships: Duration vs. Mortality (Spearman ρ = −0.023, p = 0.62), Duration vs. ΔMELD-3 (ρ = +0.039, p = 0.40), Duration vs. Improvement status (ρ = −0.064, p = 0.17), and Kruskal–Wallis test for ΔMELD-3 across duration categories (H = 1.60, p = 0.66). MELD-3 improvement rates remained relatively stable across duration categories (35.2%, 36.7%, 29.4%, 28.1%), as did mortality rates (10.6%, 12.0%, 13.7%, 4.7%) and mean ΔMELD-3 (+2.15, +2.44, +3.93, +2.55). The apparently favourable outcomes in the >60-day group (lowest mortality 4.7%) represent survival bias rather than treatment effect, as patients who died early could not accumulate long treatment durations. This analysis provides strong evidence that extending glutathione supplementation beyond the minimum 15-day course provides no additional clinical benefit.

A critical finding of this analysis was the absence of any significant relationship between glutathione treatment duration and clinical outcomes. Spearman correlation analysis showed no association between glutathione supplementation duration and mortality (ρ = −0.023, p = 0.62), ΔMELD-3 (ρ = +0.039, p = 0.40), or improvement status (ρ = −0.064, p = 0.17). Multivariable-adjusted odds ratios for the effect of glutathione duration were 0.997 per day for mortality (95% CI: 0.98–1.01, p = 0.67) and 0.998 per day for improvement (95% CI: 0.99–1.01, p = 0.63). Kruskal–Wallis testing across duration categories (≤21, 22–30, 31–60, >60 days) confirmed no significant differences in outcomes (p = 0.18 for ΔMELD, p = 0.38 for mortality) (Figure 3).

**FIGURE 4:**
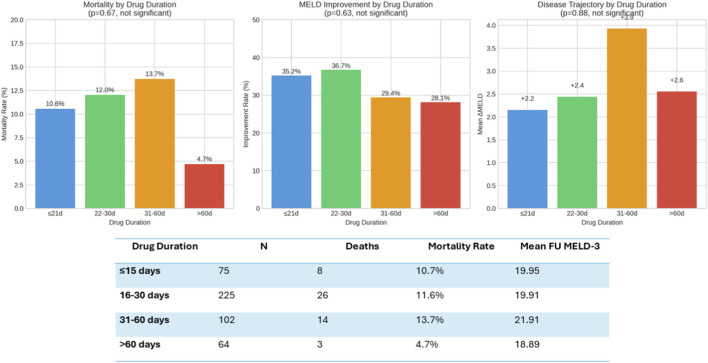
Comprehensive analysis of glutathione duration and clinical outcomes with survival bias–Three-panel analysis with accompanying summary table demonstrating the absence of dose-response effect of glutathione duration. Panel A shows mortality rates by drug duration: ≤21 days (10.6%, blue), 22–30 days (12.0%, green), 31–60 days (13.7%, orange), and >60 days (4.7%, red), with p = 0.67 (not significant). Panel B displays MELD-3 improvement rates: ≤21 days (35.2%), 22–30 days (36.7%), 31–60 days (29.4%), and >60 days (28.1%), with p = 0.63 (not significant). Panel C presents disease trajectory measured as mean ΔMELD: ≤21 days (+2.2), 22–30 days (+2.4), 31–60 days (+3.9, highest worsening), and >60 days (+2.6), with p = 0.88 (not significant). The summary table provides detailed stratification: ≤15 days (n = 75, 8 deaths, 10.7% mortality, mean follow-up MELD-3 19.95); 16–30 days (n = 225, 26 deaths, 11.6% mortality, mean follow-up MELD-3 19.91); 31–60 days (n = 102, 14 deaths, 13.7% mortality, mean follow-up MELD-3 21.91, highest); >60 days (n = 64, 3 deaths, 4.7% mortality, mean follow-up MELD-3 18.89, lowest). The apparently superior outcomes in the >60-day duration group are attributable to survival bias: only patients who survived longer could receive medication for extended durations. This comprehensive analysis confirms that glutathione treatment duration does not influence mortality, MELD-3 improvement, or disease trajectory after adjusting for confounding factors.

The apparent favourable outcomes observed in patients with longer treatment duration (>60 days: 4.7% mortality vs. 10.6%–13.7% in shorter duration groups) were attributable to survival bias rather than treatment effect. Patients who survived longer were inherently able to receive medication for longer durations. Supporting this interpretation, among long-term survivors (>365 days, alive at follow-up), shorter glutathione duration was paradoxically associated with better outcomes (≤21 days: 48% improvement vs. >60 days: 25% improvement) (Figure 4). As a sensitivity analysis to address immortal time bias, a 30-day landmark analysis was performed restricting the cohort to patients alive at day 30 (n = 419). Within this landmark cohort, glutathione treatment duration remained un-associated with ΔMELD-3 (Spearman ρ = 0.050, p = 0.31) or mortality (ρ = −0.011, p = 0.83). Duration-stratified outcomes among landmark survivors were: ≤21 days (n = 110, mortality 9.1%, improvement 37.3%, mean ΔMELD-3 +2.33), 22–30 days (n = 148, mortality 11.5%, improvement 36.5%, mean ΔMELD-3 +2.49), 31–60 days (n = 97, mortality 13.4%, improvement 30.9%, mean ΔMELD-3 +3.92), and >60 days (n = 64, mortality 4.7%, improvement 28.1%, mean ΔMELD-3 +2.55). These findings confirm that the absence of dose-response is robust to landmark conditioning.

### Higher baseline MELD-3 predicts better response

**FIGURE 5:**
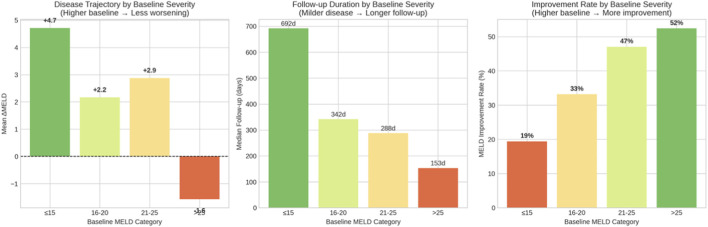
Paradoxical relationship between baseline disease severity and treatment response–Three-panel analysis demonstrating the counter-intuitive finding that higher baseline MELD-3 severity predicts better treatment response. Panel A (Disease Trajectory by Baseline Severity) shows mean ΔMELD-3 stratified by baseline MELD-3 category: ≤15 (n = 134, mean ΔMELD-3 +4.7, worst progression), 16–20 (n = 205, +2.2), 21–25 (n = 85, +2.9), and >25 (n = 42, −1.6, only group with net improvement shown in orange/red below zero line). Higher baseline MELD-3 is associated with less disease worsening (trend annotation: “Higher baseline → Less worsening”). Panel B (Follow-up Duration by Baseline Severity) demonstrates that milder disease is associated with longer follow-up: ≤15 (median 692 days), 16–20 (342 days), 21–25 (288 days), >25 (153 days, shortest), reflecting higher competing mortality risk in advanced disease. Panel C (Improvement Rate by Baseline Severity) shows a striking linear gradient where higher baseline MELD-3 predicts progressively better improvement rates: ≤15 (19%, orange), 16–20 (33%, yellow), 21–25 (47%, light green), and >25 (52%, bright green, highest improvement rate). The annotation “Higher baseline → More improvement” highlights this paradoxical relationship. This inverse relationship between baseline severity and improvement rate suggests that glutathione supplementation preferentially benefits patients with more severe disease characterized by active systemic inflammation and acute oxidative stress, rather than patients with stable compensated cirrhosis. These findings support the hypothesis that antioxidant therapy is most effective in states of acute, reversible hepatic injury where oxidative stress is a dominant, modifiable driver of disease progression.

Counter-intuitively, patients with more severe baseline disease demonstrated significantly higher rates of MELD-3 improvement (Figure 5). Patients who improved had higher baseline MELD-3 scores (median 18.69 vs. 16.42, p < 0.001), higher baseline bilirubin (4.05 vs. 2.75 mg/dL, p < 0.001), higher baseline INR (1.83 vs. 1.54, p < 0.001), and higher baseline CRP (10.2 vs. 6.55 mg/L, p = 0.016). Improvers were also younger (57 vs. 62 years, p < 0.001) and more likely to have had prior hospitalization (39.7% vs. 22.3%, p < 0.001) or prior ICU admission (12.8% vs. 4.5%, p = 0.002). Univariate logistic regression confirmed these associations: age (OR 0.971 per year, p = 0.001), baseline MELD-3 (OR 1.102 per point, p < 0.001), baseline bilirubin (OR 1.103 per mg/dL, p < 0.001), and baseline INR (OR 2.293 per unit, p < 0.001) were all significant predictors of improvement.

#### Inflammatory markers and treatment response

**FIGURE 6:**
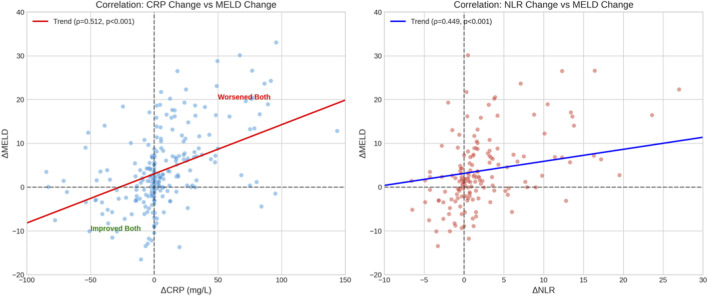
Strong correlation between inflammatory trajectory and disease progression–Scatter plots demonstrating the relationship between changes in inflammatory markers and MELD-3 score progression during glutathione treatment. Panel A shows the correlation between C-reactive protein change (ΔCRP, mg/L) and MELD-3 change (ΔMELD), with strong positive correlation (Spearman ρ = +0.512, p < 0.001, red trend line). The plot is divided into four quadrants by dashed lines at ΔCRP = 0 and ΔMELD-3 = 0, with annotations showing “Improved Both” (green, lower left quadrant where both CRP and MELD-3 decreased) and “Worsened Both” (red, upper right quadrant where both increased). Each blue circle represents one patient. The strong correlation indicates that inflammatory trajectory closely parallels disease trajectory: patients with decreasing CRP during treatment were significantly more likely to experience MELD-3 improvement, while those with rising CRP showed disease progression. Panel B displays the correlation between neutrophil-to-lymphocyte ratio change (ΔNLR) and MELD-3 change (ΔMELD), also showing significant positive correlation (Spearman ρ = +0.449, p < 0.001, blue trend line). Each salmon-coloured circle represents one patient. Similarly, increases in NLR tracked with worsening MELD-3 scores, while decreases in NLR associated with MELD-3 stabilization or improvement. These correlations provide biological plausibility for the hypothesis that glutathione acts preferentially in states of active systemic inflammation, where oxidative stress and inflammatory responses are dominant drivers of acute decompensation. The strength of these correlations (both ρ > 0.4) suggests that inflammatory biomarkers, particularly CRP trajectory, may serve as early indicators of treatment response and could guide patient selection for antioxidant therapy.

Inflammatory parameters worsened significantly during follow-up at the population level: CRP increased by +6.08 mg/L (p = 0.003), total leukocyte count by +1.43 × 10^3^/μL (p < 0.001), neutrophil-to-lymphocyte ratio (NLR) by +2.68 (p < 0.001), and absolute neutrophil count by +1.63 × 10^3^/μL (p < 0.001). Lymphocyte count decreased by −0.13 × 10^3^/μL (p = 0.016). However, changes in inflammatory markers were strongly associated with outcomes. Patients with CRP decrease during follow-up had dramatically better outcomes: 4.3% mortality versus 23.8% in those with CRP increase (p < 0.001), 52.7% improvement rate versus 16.7%, and mean ΔMELD-3 of −0.31 versus +7.79 (p < 0.001). Strong positive correlations were observed between ΔCRP and ΔMELD-3 (ρ = +0.512, p < 0.001) and between ΔNLR and ΔMELD-3 (ρ = +0.449, p < 0.001), indicating that inflammatory trajectory paralleled disease trajectory (Figure 6).

Baseline CRP ≥10 mg/L emerged as a significant predictor of MELD-3 improvement (OR 1.71, 95% CI 1.09–2.69, p = 0.025), with the effect most pronounced in patients with lower baseline MELD-3 scores (≤18), where high CRP was associated with 2.5-fold increased odds of improvement (OR 2.47, 95% CI 1.25–4.90, p = 0.014). The optimal CRP threshold by ROC analysis was 22.8 mg/L. This threshold was identified by ROC analysis on the same dataset used to test its prognostic value and is therefore subject to overfitting. It requires external validation in an independent cohort before clinical application and should be considered hypothesis-generating. More importantly, CRP trajectory during treatment was the strongest inflammatory predictor of outcomes: patients with CRP decrease showed 52.7% improvement and 4.3% mortality, compared to 23.3% improvement and 19.6% mortality in those with CRP increase (p < 0.001) (Table 3). These findings suggest that glutathione supplementation preferentially benefits patients with active inflammatory states, and that CRP monitoring may serve as a biomarker for treatment response. While baseline MELD-3 was marginally higher in patients with available CRP (median 18.0 vs. 16.4, p = 0.0008), mortality did not differ significantly (11.5% vs. 9.6%, p = 0.68), supporting the absence of meaningful selection bias for the primary endpoints.

**TABLE 3:** C-reactive protein trajectory in low MELD-3 (top) and changes during treatment (bottom) with glutathione (strongest predictor) of outcomes.

Group	N	Improvement	Mortality	Mean ΔMELD
CRP ≥10 mg/L	63	41.3%	4.8%	+2.90
CRP <10 mg/L	104	22.1%	5.8%	+3.63

Chi-square p = 0.014; OR = 2.47 (95% CI: 1.25–4.90)				
CRP trajectory	N	Improvement	Mortality	Mean ΔMELD
Major decrease (<-10)	44	61.4%	4.5%	−0.89
Moderate decrease (−10 to 0)	49	44.9%	4.1%	+0.22
Stable (0 to +10)	84	34.5%	8.3%	+2.29
Major increase (>+10)	79	11.4%	31.6%	+9.95
P Value	​	<0.001	0.001	<0.001

#### Survival analysis and predictors of mortality

Log-rank testing demonstrated significant survival differences by baseline MELD-3 category (p < 0.001): 1-year survival was 96.4% for MELD-3 ≤15, 94.6% for MELD-3 16–20, 78.3% for MELD-3 21–25, and 77.1% for MELD-3 >25. Survival also differed significantly by MELD-3 trajectory (p < 0.001), with near-perfect survival in improved/stable groups (>98%) and markedly reduced survival in deteriorated patients (74.3% at 1 year). Cox proportional hazards regression (N = 386, C-index = 0.793) identified baseline albumin as the only statistically significant independent predictor of mortality (HR 0.232, 95% CI: 0.11–0.49, p < 0.001) (Table 4). Notably, supplementation duration was not associated with survival (HR 0.995, 95% CI: 0.98–1.01, p = 0.46). In the extended Cox model including clinical complications, hepatic encephalopathy on follow-up (HR 2.128, p = 0.038) and infections on follow-up (HR 2.119, p = 0.040) emerged as significant predictors of death.

**TABLE 4:** Univariate and multivariate logistic regression analysis of predictors of survival.

Variable	Alive (median, IQR)	Died (median, IQR)	p-value
Univariate logistic regression on baseline predictors			
Age (years)	60.0 (52.0–68.0)	62.0 (56.0–66.5)	0.295
Follow-up duration (days)	445.0 (140.0–791.0)	208.0 (84.0–529.0)	0.003
Baseline haemoglobin (g/dL)	11.7 (10.5–13.1)	11.1 (9.6–12.5)	0.008
Baseline albumin (g/dL)	3.1 (2.7–3.6)	2.7 (2.5–2.9)	<0.001
Baseline INR	1.63 (1.35–1.97)	1.84 (1.50–2.23)	0.01
Baseline MELD-3	16.4 (14.0–20.0)	20.4 (16.4–22.7)	<0.001
Baseline ALBI	−1.48 (−1.90 to −1.08)	−1.04 (−1.48 to −0.81)	<0.001
Univariate logistic regression on follow-up predictors			
Follow-up haemoglobin (g/dL)	11.4 (9.2–13.0)	8.6 (7.1–11.0)	<0.001
Follow-up total bilirubin (mg/dL)	3.3 (1.6–5.4)	12.9 (4.5–19.9)	<0.001
Follow-up albumin (g/dL)	3.0 (2.5–3.5)	2.4 (2.1–2.7)	<0.001
Follow-up INR	1.65 (1.40–2.13)	3.52 (2.12–4.57)	<0.001
Follow-up CRP (mg/L)	8.5 (4.0–23.9)	34.9 (20.3–71.1)	<0.001
Follow-up MELD-3	16.8 (14.9–21.2)	34.2 (28.4–39.5)	<0.001
Follow-up ALBI	−1.31 (−1.71 to −0.99)	−0.55 (−0.81 to −0.36)	<0.001

Multivariate logistic regression - model: Baseline predictors (N = 406)			
Variable	Adjusted OR	95% CI	p-value
Age	1.024	0.99–1.06	0.1370
Baseline MELD-3	1.035	0.95–1.12	0.4160
Baseline ALBI	0.550	0.07–4.19	0.5642
Baseline albumin	0.138	0.02–0.99	0.0486

Model Summary: Pseudo *R* ^2^ = 0.094, AIC, 269.98. Key Finding: In multivariate analysis, baseline albumin remained the only independent predictor of mortality, with each 1 g/dL increase associated with 86% lower odds of death (OR, 0.14).

##### Machine learning feature importance

Random Forest modelling for mortality prediction achieved a cross-validated AUC of 0.625 ± 0.091. The most important features were baseline MELD-3 (importance: 0.118), baseline INR (0.095), haemoglobin (0.094), age (0.092), and bilirubin (0.085). Drug duration ranked low in importance (0.054), consistent with the absence of dose-response relationship observed in conventional analyses. For MELD-3 improvement prediction, Random Forest achieved a cross-validated AUC of 0.670 ± 0.068. Top predictors were baseline MELD-3 (0.122), baseline INR (0.102), baseline bilirubin (0.099), age (0.099), and platelet count (0.094). Again, drug duration contributed minimally (0.063). The modest discriminative performance of the Random Forest models (AUC 0.625 for mortality, 0.670 for improvement) should be interpreted in context. These models were developed not as clinical prediction tools but as a complementary method for ranking feature importance. The low AUC values likely reflect the fact that baseline variables alone capture only a fraction of outcome variance in chronic liver disease, where intercurrent events during follow-up (infections, variceal bleeding, acute kidney injury) are dominant determinants. Similarly, the low Nagelkerke pseudo-R^2^ (0.094) of the baseline logistic regression model reflects the inherent unpredictability of cirrhosis outcomes from baseline parameters alone and underscores that follow-up trajectory, rather than any single baseline measurement, is the primary driver of prognosis.

#### Cluster analysis: Identification of patient phenotypes

K-means clustering identified four distinct patient phenotypes with markedly different clinical profiles and outcomes:

*Cluster 0* – ‘Potential Poor Responders’ (N = 117, 25.1%): This cluster had the lowest baseline MELD-3 (12.1), highest albumin (3.80 g/dL), and lowest bilirubin (1.70 mg/dL). Patients were older (median 65 years) and predominantly had MASLD (65.8%). Despite excellent survival (3.4% mortality), improvement rates were low (25.6%) with mean ΔMELD-3 of +3.44, suggesting limited benefit from glutathione supplementation in compensated disease.

*Cluster 1* – ‘Potential Intermediate Responders’ (N = 255, 54.7%): The largest cluster had moderate MELD-3 (18.1), low albumin (2.84 g/dL), and elevated bilirubin (4.18 mg/dL). Etiology was mixed (46.3% MASLD, 41.2% ALD). Outcomes were moderate: 12.2% mortality, 30.2% improvement, and mean ΔMELD-3 of +3.19.

*Cluster 2* – ‘Potential Early Responders’ (N = 43, 9.2%): This cluster showed moderate MELD-3 (16.8), preserved albumin (3.00 g/dL), and older age (median 65 years). Despite high HCC prevalence (34.9%), this group demonstrated excellent improvement rates (51.2%) with low mortality (9.3%).

*Cluster 3* – ‘Potential High-Risk Responders’ (N = 51, 10.9%): This phenotype had the highest baseline MELD-3 (27.9), lowest albumin (2.57 g/dL), and highest bilirubin (10.59 mg/dL). Patients were youngest (median 53 years) and predominantly had ALD (52.9%). Remarkably, this cluster showed the highest improvement rate (52.9%) and was the only cluster with mean MELD-3 improvement (ΔMELD-3 = −0.85). However, mortality was also highest (23.5%), reflecting the ‘high-risk, high reward’ nature of this phenotype.

Cluster validation metrics for the four-cluster solution showed modest geometric separation in input feature space: silhouette score 0.141, Davies-Bouldin index 1.833, and Calinski-Harabasz index 48.6. These values indicate substantial overlap between clusters and confirm that the partition is not a clean separation of the cohort; this is consistent with the continuous, multidimensional nature of cirrhosis severity, in which most patients lie along feature gradients rather than within discrete classes. The cluster solution is therefore presented as an exploratory data-reduction and hypothesis-generating framework, not as a validated diagnostic taxonomy. Two findings nonetheless support its descriptive utility for the present analysis. First, stability across 20 random seed initialisations yielded a mean Adjusted Rand Index of 0.843 (range 0.466–1.000), indicating that the same broad partition is recovered reproducibly despite the low silhouette—the silhouette reflects between-cluster overlap, not the reproducibility of cluster assignment. Second, the clusters were separated by large and highly significant differences in outcome variables (mortality, ΔMELD-3, improvement rate) that were not used as inputs to the clustering, providing clinical face validity that the geometric silhouette metric does not capture. We make no claim that this partition is unique, optimal, or externally generalisable. The labels assigned to each cluster describe central tendencies in baseline features only; external replication in independent cohorts, using both k-means and alternative methods (hierarchical clustering, Gaussian mixture models, latent class analysis), is required before any clinical use of this framework.

#### Ideal responder profile

**FIGURE 7:**
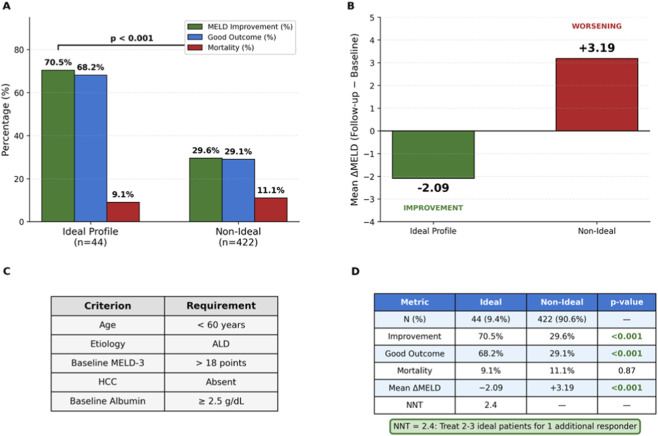
Clinical characteristics and outcomes of the ideal responder profile–Comprehensive four-panel analysis defining and characterizing patients most likely to benefit from glutathione supplementation. **(A)** (Clinical Outcomes by Responder Profile) compares the “Ideal Profile” (n = 44, 9.4% of cohort) versus “Non-Ideal” profile (n = 422) across three outcomes: MELD-3 improvement rate (70.5% vs. 29.6%, green bars, p < 0.001), Good outcome rate (68.2% vs. 29.1%, blue bars, defined as survival with stable/improved MELD), and Mortality (9.1% vs. 11.1%, red bars). The dramatic difference in improvement rates demonstrates the clinical utility of this patient selection strategy. **(B)** (MELD-3 Score Change) shows mean ΔMELD-3 for Ideal Profile (−2.09, green bar below zero line, labelled “IMPROVEMENT”) compared to non-ideal profile (+3.19, red bar above zero line, labelled “WORSENING”), representing a 5.28-point difference in disease trajectory (p < 0.001). **(C)** (Ideal Responder Profile Criteria) presents the selection criteria in tabular format: Age <60 years, Etiology = ALD (alcohol-associated liver disease), Baseline MELD-3 >18 points, HCC = Absent, and Baseline Albumin ≥2.5 g/dL. These criteria identify younger patients with alcohol-related disease, elevated baseline MELD-3 indicating active disease, preserved synthetic function, and absence of malignancy. **(D)** (Summary Statistics) provides detailed comparative outcomes: sample sizes (44 vs. 422), improvement rates (70.5% vs. 29.6%, p < 0.001), good outcome rates (68.2% vs. 29.1%, p < 0.001), mortality (9.1% vs. 11.1%, p = 0.87, not significant), mean ΔMELD-3 (−2.09 vs. +3.19, p < 0.001), and Number Needed to Treat (NNT = 2.4, shown in green box at bottom). The NNT of 2.4 indicates that treating two to three patients matching the ideal profile would result in one additional patient achieving MELD-3 improvement compared to the non-ideal population. This profile identifies a biologically distinct subgroup where oxidative stress and systemic inflammation are likely dominant, modifiable drivers of acute decompensation, providing a precision-medicine framework for rational use of glutathione in chronic liver disease.

Based on subgroup analyses, a potentially ‘Ideal Responder Profile’ was identified comprising patients with age <60 years, ALD etiology, baseline MELD-3 > 18, absence of HCC, and baseline albumin ≥2.5 g/dL. This profile, present in 9.4% of patients (n = 44), was associated with dramatically superior outcomes: 70.5% improvement rate (vs. 29.6% in non-ideal profile), mean ΔMELD-3 of −2.09 (actual MELD-3 improvement, vs. +3.19 worsening), and 68.2% good outcome rate (vs. 29.1%). The comparison was highly significant (p < 0.0001), with a number needed to treat (NNT) of 2.4 for one additional patient to achieve MELD-3 improvement (Figure 7).

Significant heterogeneity in treatment response was observed across liver disease etiologies. ALD patients showed the highest improvement rate (38.8%) with the most favourable ΔMELD-3 trajectory (+1.65), while MASLD patients showed lower improvement (28.9%) and greater disease progression (+3.59). Acute viral hepatitis (AVH) was the only group with mean MELD-3 improvement (ΔMELD-3 = −1.18), and DILI patients showed 50% improvement rate. Interaction analysis revealed that ALD patients showed progressively better response with increasing baseline severity (15.6% improvement in MELD-3 ≤15 vs. 57.9% in MELD-3 21–25), while this gradient was less pronounced in MASLD.

### Composite outcomes

Good outcome (survival with stable/improved MELD) was achieved by 32.8% of patients (n = 153). Poor outcome (death or ΔMELD-3 > 10) occurred in 17.4% (n = 81). Clinical success (survival without major complications) was observed in 49.6% (n = 231). The ‘Potential Early Responders’ had the highest clinical success rate (65.0%), while the ‘Potential High-Risk Responders’ cluster had the lowest (33.3%).

## Discussion

This retrospective single-arm analysis of 466 patients with chronic liver disease receiving oral glutathione supplementation describes several patterns that, while not establishing treatment efficacy, are relevant to how antioxidant therapy is conceptualised and prescribed in cirrhosis. First, we found no evidence of a dose-response relationship between glutathione treatment duration and clinical outcomes. Second, we discovered a paradoxical relationship between baseline disease severity and treatment response, wherein patients with more severe disease showed higher rates of improvement. Third, we identified distinct patient phenotypes with markedly different treatment responses, enabling practical applications in the clinic.

Hepatic glutathione depletion is a consistent feature of ALD and MASLD and contributes to oxidative stress, mitochondrial dysfunction, and disease progression. This pathophysiological rationale has underpinned the use of glutathione, N-acetylcysteine and related strategies in chronic liver disease. However, human data are sparse. The most cited clinical trial is the open-label MASLD pilot by Honda et al., in which 300 mg/day oral glutathione for 4 months reduced ALT and hepatic steatosis in 29 patients, but without histological or survival data and with no control arm (Honda et al., 2017). A critical pharmacological consideration is the bioavailability of oral glutathione. Intact glutathione is substantially degraded by intestinal γ-glutamyltranspeptidase and intraluminal peptidases, and early studies suggested negligible systemic bioavailability following oral administration. However, more recent data have demonstrated that oral glutathione (250–1,000 mg/day) can raise plasma and erythrocyte GSH levels in healthy volunteers and reduce oxidative stress markers, possibly through absorption of intact glutathione via intestinal epithelial transport or through provision of cysteine as a rate-limiting precursor for intracellular GSH synthesis. Whether 500 mg/day achieves pharmacologically meaningful hepatic target engagement in patients with cirrhosis, where intestinal permeability, portal hypertension, and first-pass metabolism are all altered, remains unknown and is a significant uncertainty underlying the biological interpretation of this study. Subsequent reviews have concluded that evidence for glutathione in MASLD and other chronic liver diseases remains preliminary, limited by small sample sizes, short follow-up, and surrogate endpoints (Santacroce et al., 2023). Against this background, the current cohort provides a much more sobering picture. In an unselected, predominantly cirrhotic population with a median MELD-3 of 16.4, oral glutathione was associated with net MELD-3 worsening, rising bilirubin and INR, and only 32.8% achieving the composite “good outcome” of survival with stable/improved MELD. These findings argue strongly against a clinically meaningful disease-modifying effect of low-dose oral glutathione when prescribed indiscriminately to patients with chronic liver disease.

The impact of glutathione supplementation on this cohort presents a complex picture that requires careful interpretation. At the population level, the overall trajectory was one of disease progression rather than improvement: the mean MELD-3 score increased by 2.69 points from baseline to follow-up along with statistically significant changes indicating hepatic decompensation. The complication rates also increased substantially during follow-up. However, it would be scientifically inappropriate to attribute this progression directly to glutathione supplementation, as these patients had advanced chronic liver disease with a natural trajectory toward decompensation, and critically, there was no control group to compare against.

What the data does reveal additionally is that approximately one-third of patients experienced MELD-3 improvement during follow-up, and these patients had remarkably low mortality. The 33.5% improvement rate with associated 1.9% mortality is noteworthy, particularly given that these patients had higher baseline disease severity (median MELD-3 18.7) compared to those who worsened (median MELD-3 16.4). Another 23.6% remained stable with very low mortality. This combined 57.1% of patients with stable or improved disease had a mortality rate of only 1.5% which is exceptionally low for a population with baseline median MELD-3 of 16.4. This suggests that while the overall population showed disease progression, a substantial subset either stabilized or improved, and their outcomes were excellent. This paradox–that patients with higher baseline severity showed higher rates of MELD-3 improvement–is compatible with several mechanisms, including (but not limited to) a possible therapeutic effect of glutathione in inflammation-rich states, control of the precipitating event by concurrent standard care, and statistical regression toward the mean; the present single-arm design cannot distinguish between these possibilities. Patients with higher baseline bilirubin, higher baseline INR, and higher MELD-3 scores showed greater propensity for improvement, which may indicate that glutathione’s antioxidant properties are more beneficial in states of a phenotype akin to “acutely worsened cirrhosis state”, where oxidative stress was highest. Additionally, the mortality rate of 10.9% over a median follow-up of 417 days is relatively favourable for this patient population. Historical cohorts of decompensated cirrhosis patients with similar MELD-3 scores typically experience 20%–30% annual mortality. The extended drug duration subgroup (>60 days) showed numerically lower mortality (4.7% vs. 10.7%–13.7% in shorter-duration groups) and the lowest mean follow-up MELD-3 score (18.89 vs. ∼20–22 in other groups); however, as already discussed and confirmed by the 30-day landmark analysis, this apparent advantage may be related to survival bias.

The finding that follow-up parameters vastly outperformed baseline parameters in predicting mortality (Follow-up MELD-3 AUC = 0.912 vs. Baseline MELD-3 AUC = 0.650) suggests that disease trajectory during treatment, rather than treatment itself, is the dominant factor determining outcomes. Perhaps the most clinically actionable finding is the strong association between disease trajectory and outcomes. Patients with MELD-3 improvement or stability showed near-perfect survival (>98% at 1 year), while those with severe deterioration had only 74% survival. This validates serial MELD-3 monitoring as the primary method for assessing treatment response and informs clinical decision-making. This mirrors studies in transplant and non-transplant populations where increases of ≥5–10 MELD-3 points over weeks to months substantially increase short-term mortality risk (D'Amico, 2005). The current data therefore reinforce MELD-3 change as a valid surrogate of disease trajectory and confirm that any putative benefit of glutathione would have to be visible at the level of MELD-3 dynamics to be clinically meaningful–but it is not.

Baseline serum albumin emerged as the only independent predictor of mortality in the main Cox model, and infections and hepatic encephalopathy during follow-up were strong predictors in the extended model. This pattern is entirely in line with large contemporary cohorts showing albumin-bilirubin (ALBI) indices and hypoalbuminemia as powerful markers of hepatic reserve and survival in cirrhosis, including ICU and sepsis settings (Wang et al., 2025). Similarly, multiple studies have shown that infections increase mortality roughly four-fold in cirrhotic patients and that hepatic encephalopathy independently predicts death and repeated hospitalisation (Arvaniti et al., 2010; Riggio et al., 2023). The present study adds little that is reassuring: glutathione did not mitigate the impact of these classic complications, and its duration was not associated with survival once baseline albumin and clinical events were accounted for.

The most intriguing, and potentially important finding is the clear link between inflammatory burden, inflammatory trajectory, and likelihood of MELD-3 improvement. Baseline CRP ≥10 mg/L predicted higher odds of MELD-3 improvement, particularly in those with MELD-3 ≤18, and decreases in CRP and NLR during follow-up were strongly associated with MELD-3 improvement, lower ΔMELD, and substantially lower mortality. In contrast, rising CRP and NLR tracked closely with disease progression. These observations align closely with the now-dominant view of cirrhosis and ACLF as inflammation-driven syndromes in which systemic inflammatory response, rather than static structural damage alone, determines short-term prognosis (Moreau et al., 2013). CRP and NLR are established prognostic markers in decompensated cirrhosis and ACLF; higher baseline values and failure to normalise predict early mortality (Cervoni et al., 2012). The present cohort goes further by demonstrating that on-treatment CRP trajectory has stronger prognostic value than baseline CRP and correlates tightly with changes in MELD.

The observation that patients with higher baseline MELD-3, bilirubin, INR, and CRP showed higher rates of MELD-3 improvement is biologically interesting. It has previously been hypothesised, on mechanistic grounds, that such patients are in a state of acute reversible oxidative injury rather than chronic persistent inflammation, and that exogenous glutathione might transiently restore intracellular redox capacity in this window. Within that hypothetical framework, patients with acute decompensation or an ACLF-like phenotype could be conceived of as experiencing a burst of reactive oxygen species generation that overwhelms endogenous antioxidant defences, with exogenous glutathione repletion mechanistically plausible during a transient ‘window of vulnerability’. Recent experimental work consistent with this framework includes studies of CYP2E1-mediated oxidative stress in alcohol-related liver injury and of stage-dependent oxidative responses in repeated toxic liver injury (Jung et al., 2024; Hammad et al., 2023). We wish to emphasise, however, that the present study did not measure any direct marker of hepatic or systemic oxidative stress—such as the GSH/GSSG ratio, malondialdehyde, F2-isoprostanes, or 8-hydroxy-2′-deoxyguanosine—and the observed clinical pattern is equally compatible with several non-redox explanations: differential reversibility of the underlying precipitating event between phenotypes, differential intensity of concurrent standard-of-care interventions in the sicker subgroup (infection control, alcohol abstinence, diuretic optimisation, nutritional support), and statistical regression toward the mean in patients with the highest baseline biochemical values. The redox-threshold framework is therefore offered as a biologically plausible hypothesis to be tested in a prospective trial that incorporates direct oxidative stress biomarkers, and not as a mechanistic claim supported by the data of this study.

**FIGURE 8:**
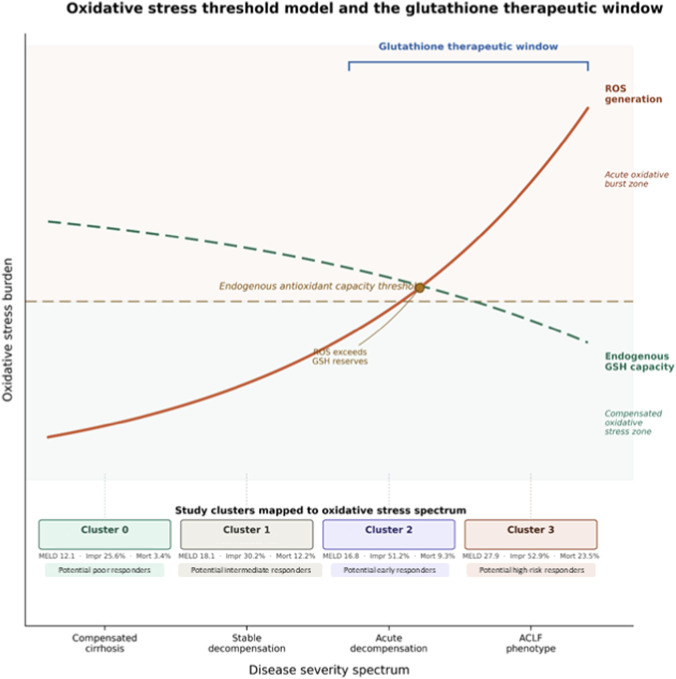
Proposed conceptual model of an oxidative-stress threshold and a putative glutathione therapeutic window in chronic liver disease–This figure depicts a biologically plausible hypothesis, not a finding of the present study; no direct redox biomarker was measured in this cohort. As disease severity progresses from compensated cirrhosis to acute-on-chronic liver failure (ACLF), reactive oxygen species (ROS) generation increases exponentially (solid red curve) while endogenous glutathione (GSH) capacity declines progressively (dashed green curve). In compensated and stable decompensated cirrhosis (lower-left region), endogenous antioxidant reserves maintain redox equilibrium, and exogenous glutathione supplementation provides minimal additional benefit. Beyond the antioxidant capacity threshold (dashed brown line) — where ROS generation exceeds endogenous GSH reserves (crossing point, brown circle) — a transient therapeutic window emerges (blue bracket) in which exogenous glutathione may achieve pharmacologically relevant redox modulation. The four patient phenotypes identified by K-means cluster analysis are mapped to their corresponding positions along this spectrum.

That said, it is tempting, but not yet defensible, to infer a causal link between glutathione administration, dampening of inflammation, and MELD-3 improvement in the subgroup with CRP reduction (Figure 8). This study lacks a control group, and CRP change is likely influenced by standard care (infection treatment, diuresis, abstinence from alcohol, nutritional support) at least as much as by glutathione *per se*. Nevertheless, the convergence of (i) higher baseline CRP among improvers, (ii) the strong correlation with ΔCRP/ΔNLR and ΔMELD, and (iii) the recognised role of glutathione in modulating redox-sensitive inflammatory pathways, does support a biologically coherent hypothesis: glutathione may be most useful in patients with an “inflammatory” phenotype, where oxidative stress and systemic inflammation are dominant, modifiable drivers of acute decompensation (Vairetti et al., 2021). Nonetheless, a notable limitation of the inflammatory biomarkers used in this study is their lack of specificity for oxidative stress. CRP and NLR are markers of systemic inflammation broadly, and their trajectory during follow-up may reflect resolution of infections, alcohol abstinence, or other standard-of-care interventions rather than a direct antioxidant effect of glutathione. Future studies should incorporate oxidative stress-specific biomarkers such as malondialdehyde, F2-isoprostanes, 8-hydroxy-2′-deoxyguanosine, or the GSH/GSSG ratio to determine whether glutathione supplementation achieves measurable target engagement at the level of hepatic redox biology.

The apparent counter-intuitive finding that patients with higher baseline MELD, bilirubin, INR and CRP were more likely to improve also fits this picture. High-MELD-3 patients have more headroom for regression toward the mean and are over-represented among those with acute inflammatory flares on a background of chronic disease. Studies of decompensated cirrhosis and ACLF consistently show that patients with intense systemic inflammation can improve dramatically if the precipitating event is controlled but have high competing mortality risk (Saeidinejad et al., 2023). Our results validate the same–the “Potential High-Risk Responder” cluster (cluster 3) had the highest baseline MELD-3 and inflammatory burden, the only negative mean ΔMELD-3 (improvement), and the highest mortality. The four phenotypes identified by k-means clustering map logically onto recognised clinical spectra of cirrhosis–compensated/early disease, stable decompensation, treatment-responsive unstable disease, and advanced decompensation. Comparable data-driven phenotyping in cirrhosis and ACLF has repeatedly highlighted inflammation-rich clusters with both high short-term mortality and high potential for rapid improvement when reversibility is present (Laleman et al., 2018). The current study adds the observation that glutathione-treated patients in the “Potential High-Risk Responder/ALD-predominant” cluster showed the only net MELD-3 improvement at the group level yet remained at highest absolute risk of death–the classic “high-risk, high-reward” biology. In other words, in patients already on a relatively benign trajectory, adding glutathione did not meaningfully change the MELD-3 slope.

The preferential response in ALD and in patients younger than 60 years with higher baseline MELD, preserved albumin (≥2.5 g/dL) and no HCC–defining the “ideal responder profile” – is biologically plausible. Alcohol-related disease is characterised by profound oxidative stress, glutathione depletion, and a prominent inflammatory component driven by gut-derived endotoxin and cytokines. Younger age, preserved synthetic function, and absence of HCC identify those with enough hepatic reserve for any antioxidant/inflammation-modulating intervention to translate into measurable clinical recovery. But it must be interpreted with caution: without a control arm, we cannot exclude that this profile simply marks patients who would have improved with optimal standard care alone. The ideal responder profile, while biologically coherent, was identified through *post hoc* subgroup exploration and is vulnerable to overfitting and multiple comparison artefacts. It is presented as a hypothesis to be tested, ideally in a prospective, enrichment-design trial, and not as a validated patient selection tool. The contrasting poorer response in MASLD, where improvement rates were lower and ΔMELD-3 more unfavourable, is also consistent with the literature. In NAFLD/MASLD, antioxidant approaches (vitamin E, glutathione) show modest biochemical and histological benefit mainly in earlier, non-cirrhotic stages, but robust outcome data in advanced disease are lacking (Sumida and Yoneda, 2018). Once portal hypertension and decompensation are established, systemic metabolic and cardiovascular comorbidities become major competing risks that simple antioxidant replenishment is unlikely to overcome.

Our finding that treatment duration did not influence outcomes across multiple analytic approaches (correlation analysis, stratified analysis, multivariate regression, machine learning) has important clinical implications. This would suggest that extended glutathione supplementation provides no additional benefit and may represent unnecessary healthcare resource utilization. The superficially better crude outcomes in patients treated for >60 days are convincingly explained by survival bias: only those who survived and remained clinically stable could accumulate long treatment durations. The observation that, among long-term survivors (>365 days), shorter glutathione courses were associated with better MELD-3 outcomes is itself subject to selection bias (conditioning on survival selects for favourable trajectories), and a formal 30-day landmark analysis was therefore performed, which confirmed the absence of any duration–outcome relationship. These data directly challenge any assumption that “more glutathione is better” and are concordant with the broader literature, in which escalating or prolonged antioxidant therapy has rarely translated into clear survival benefit in cirrhosis.

### Study limitations

Several limitations should be acknowledged. First, this is a retrospective, single-center study without a control group, precluding definitive conclusions about treatment efficacy versus natural disease progression. No causal inferences about glutathione efficacy can be drawn. All observed associations between patient characteristics and MELD-3 trajectory may reflect natural disease course, concurrent standard-of-care interventions, or regression to the mean rather than any pharmacological effect of glutathione. Second, survival bias affects the interpretation of treatment duration effects, as patients who died could not complete longer treatment courses. Third, missing data (13%–45% for some laboratory parameters) may introduce bias, though we employed complete-case analysis with sensitivity analyses. Fourth, the mechanism of action remains speculative and requires laboratory confirmation. Fifth, we could not assess medication adherence or concurrent therapies that may have influenced outcomes. Sixth, the cohort was predominantly male (85.2%), from a single tertiary care centre with MASLD and ALD as predominant etiologies. The findings may not be generalizable to populations where viral hepatitis, autoimmune hepatitis, or cholestatic liver disease predominate, or to healthcare settings with different standard-of-care protocols, alcohol consumption patterns, or nutritional backgrounds. The high HCC prevalence also reflects the tertiary referral nature of this centre. Multicenter validation across diverse geographies and etiologic profiles is needed.

## Conclusion

Taken together, these findings do not support routine oral glutathione supplementation as a disease-modifying therapy in unselected patients with chronic liver disease. The cohort as a whole continued to progress biochemically, and duration of therapy had no detectable impact on MELD-3 trajectory or survival once confounding was addressed. At the same time, the data were not purely negative. They hint at a narrow therapeutic window in a biologically distinct subgroup: younger patients, usually with alcohol-related disease, higher baseline MELD-3 but preserved albumin, and clear evidence of systemic inflammation (higher CRP) in whom improvements in inflammatory markers parallel meaningful improvements in MELD-3 and outcomes. This is exactly the phenotype in which targeting oxidative stress and inflammation is conceptually most rational.

The appropriate conclusion is therefore not that “glutathione works” in this group, but that this is the only group in which it might be worth studying. The next logical step would be a prospective, biomarker-stratified, randomised trial in patients with decompensated ALD with evidence of high systemic inflammation, enriched for high CRP/NLR and preserved albumin, with glutathione versus placebo on top of standard care, and with MELD-3 trajectory, CRP change and pragmatic clinical outcomes as endpoints. Parallel measurement of redox biomarkers would be essential to demonstrate on-target pharmacodynamic effects. Until such data exist, the current results should be regarded as hypothesis-generating and a useful warning against indiscriminate use of glutathione in chronic liver disease.

## Data Availability

The original contributions presented in the study are included in the article/Supplementary Material, further inquiries can be directed to the corresponding author.

## References

[B1] Arvaniti V. D'Amico G. Fede G. Manousou P. Tsochatzis E. Pleguezuelo M. (2010). Infections in patients with cirrhosis increase mortality four-fold and should be used in determining prognosis. Gastroenterology 139 (4), 1246–1256. 10.1053/j.gastro.2010.06.019 20558165

[B2] Bjelakovic G. Gluud L. L. Nikolova D. Bjelakovic M. Nagorni A. Gluud C. (2010). Meta-analysis: antioxidant supplements for liver diseases - the cochrane hepato-biliary group. Aliment. Pharmacol. Ther. 32 (3), 356–367. 10.1111/j.1365-2036.2010.04371.x 20497142

[B3] Cervoni J. P. Thévenot T. Weil D. Muel E. Barbot O. Sheppard F. (2012). C-reactive protein predicts short-term mortality in patients with cirrhosis. J. Hepatol. 56 (6), 1299–1304. 10.1016/j.jhep.2011.12.030 22314431

[B4] D'Amico G. (2005). Developing concepts on MELD: delta and cutoffs. J. Hepatol. 42 (6), 790–792. 10.1016/j.jhep.2005.03.009 15885348

[B5] Engelmann C. Clària J. Szabo G. Bosch J. Bernardi M. (2021). Pathophysiology of decompensated cirrhosis: portal hypertension, circulatory dysfunction, inflammation, metabolism and mitochondrial dysfunction. J. Hepatol. 75 (Suppl. 1), S49–S66. 10.1016/j.jhep.2021.01.002 34039492 PMC9272511

[B6] Hammad S. Ogris C. Othman A. Erdoesi P. Schmidt-Heck W. Biermayer I. (2023). Tolerance of repeated toxic injuries of murine livers is associated with steatosis and inflammation. Cell. Death Dis. 14 (7), 414. 10.1038/s41419-023-05855-4 37438332 PMC10338629

[B7] Honda Y. Kessoku T. Sumida Y. Kobayashi T. Kato T. Ogawa Y. (2017). Efficacy of glutathione for the treatment of nonalcoholic fatty liver disease: an open-label, single-arm, multicenter, pilot study. BMC Gastroenterol. 17 (1), 96. 10.1186/s12876-017-0652-3 28789631 PMC5549431

[B8] Jung Y. S. Radhakrishnan K. Hammad S. Müller S. Müller J. Noh J. R. (2024). ERRγ-inducible FGF23 promotes alcoholic liver injury through enhancing CYP2E1 mediated hepatic oxidative stress. Redox Biol. 71, 103107. 10.1016/j.redox.2024.103107 38479224 PMC10950689

[B9] Kronsten V. T. Shawcross D. L. (2025). Clinical implications of inflammation in patients with cirrhosis. Am. J. Gastroenterol. 120 (1), 65–74. 10.14309/ajg.0000000000003056 39194320 PMC11676607

[B10] Laleman W. Claria J. Van der Merwe S. Moreau R. Trebicka J. (2018). Systemic inflammation and acute-on-chronic liver failure: too much, not enough. Can. J. Gastroenterol. Hepatol. 2018, 1027152. 10.1155/2018/1027152 30155448 PMC6093057

[B11] Moreau R. Jalan R. Gines P. Pavesi M. Angeli P. Cordoba J. (2013). Acute-on-chronic liver failure is a distinct syndrome that develops in patients with acute decompensation of cirrhosis. Gastroenterology 144 (7), 1426–1437. 10.1053/j.gastro.2013.02.042 23474284

[B12] Mynster K. T. Webel H. O'Connell M. B. Danielsen K. V. Hobolth L. Møller S. (2023). Markers of inflammation predict survival in newly diagnosed cirrhosis: a prospective registry study. Sci. Rep. 13 (1), 20039. 10.1038/s41598-023-47384-2 37973887 PMC10654496

[B13] Riggio O. Celsa C. Calvaruso V. Merli M. Caraceni P. Montagnese S. (2023). Hepatic encephalopathy increases the risk for mortality and hospital readmission in decompensated cirrhotic patients: a prospective multicenter study. Front. Med. (Lausanne) 10, 1184860. 10.3389/fmed.2023.1184860 37305121 PMC10248517

[B14] Sacco R. Eggenhoffner R. Giacomelli L. (2016). Glutathione in the treatment of liver diseases: insights from clinical practice. Minerva Gastroenterol. Dietol. 62 (4), 316–324. 27603810

[B15] Saeidinejad M. Elshabrawi A. Sriphoosanaphan S. Andreola F. Mehta G. Agarwal B. (2023). Novel therapeutic approaches in treatment of acute-on-chronic liver failure. Semin. Liver Dis. 43 (4), 429–445. 10.1055/s-0043-1776773 38101419 PMC10723941

[B16] Santacroce G. Gentile A. Soriano S. Novelli A. Lenti M. V. Di Sabatino A. (2023). Glutathione: pharmacological aspects and implications for clinical use in non-alcoholic fatty liver disease. Front. Med. (Lausanne) 10, 1124275. 10.3389/fmed.2023.1124275 37035339 PMC10075255

[B17] Singal A. K. Jampana S. C. Weinman S. A. (2011). Antioxidants as therapeutic agents for liver disease. Liver Int. 31 (10), 1432–1448. 10.1111/j.1478-3231.2011.02604.x 22093324 PMC3228367

[B18] Sumida Y. Yoneda M. (2018). Current and future pharmacological therapies for NAFLD/NASH. J. Gastroenterol. 53 (3), 362–376. 10.1007/s00535-017-1415-1 29247356 PMC5847174

[B19] Trebicka J. Amoros A. Pitarch C. Titos E. Alcaraz-Quiles J. Schierwagen R. (2019). Addressing profiles of systemic inflammation across the different clinical phenotypes of acutely decompensated cirrhosis. Front. Immunol. 10, 476. 10.3389/fimmu.2019.00476 30941129 PMC6434999

[B20] Vairetti M. Di Pasqua L. G. Cagna M. Richelmi P. Ferrigno A. Berardo C. (2021). Changes in glutathione content in liver diseases: an update. Antioxidants (Basel) 10 (3), 364. 10.3390/antiox10030364 33670839 PMC7997318

[B21] Wang J. Yang P. Qin C. Huang Y. Hu Z. Shi R. (2025). Predictive role of the albumin-bilirubin score in ICU patients with cirrhosis and sepsis: insights from a large retrospective cohort. BMC Gastroenterol. 25 (1), 520. 10.1186/s12876-025-04111-7 40665253 PMC12261734

