# Supplementary material for: A cluster analysis of 466 patients demonstrates that glutathione supplementation could preferentially benefit advanced unstable cirrhosis phenotype rather than stable cirrhosis
Source: Front Pharmacol. 2026 Jun 30;17:1767206. doi: 10.3389/fphar.2026.1767206 (PMC13364553; doi:10.3389/fphar.2026.1767206)
Supplement: Supplementary file 1 [file Table1.DOCX]

**Supplementary Table1:** Missingness rates per variable

| Variable | Missing n (%) |
| --- | --- |
| Baseline Haemoglobin | 73 (15.7%) |
| Baseline TLC | 73 (15.7%) |
| Baseline NLR | 193 (41.4%) |
| Baseline Platelet | 73 (15.7%) |
| Baseline Total Bilirubin | 60 (12.9%) |
| Baseline Albumin | 60 (12.9%) |
| Baseline INR | 79 (17.0%) |
| Baseline Creatinine | 70 (15.0%) |
| Baseline Sodium | 110 (23.6%) |
| Baseline CRP | 135 (29.0%) |
| Baseline MELD-3 | 0 (0.0%) |
| Baseline ALBI | 0 (0.0%) |
| Follow-up Haemoglobin | 109 (23.4%) |
| Follow-up TLC | 110 (23.6%) |
| Follow-up NLR | 223 (47.9%) |
| Follow-up Platelet | 112 (24.0%) |
| Follow-up Total Bilirubin | 91 (19.5%) |
| Follow-up Albumin | 93 (20.0%) |
| Follow-up INR | 114 (24.5%) |
| Follow-up Creatinine | 98 (21.0%) |
| Follow-up Sodium | 117 (25.1%) |
| Follow-up CRP | 141 (30.3%) |
| Follow-up MELD-3 | 0 (0.0%) |
| Follow-up ALBI | 0 (0.0%) |
