# Supplementary figures and images for: A cluster analysis of 466 patients demonstrates that glutathione supplementation could preferentially benefit advanced unstable cirrhosis phenotype rather than stable cirrhosis
Source: Front Pharmacol. 2026 Jun 30;17:1767206. doi: 10.3389/fphar.2026.1767206 (PMC13364553; doi:10.3389/fphar.2026.1767206)

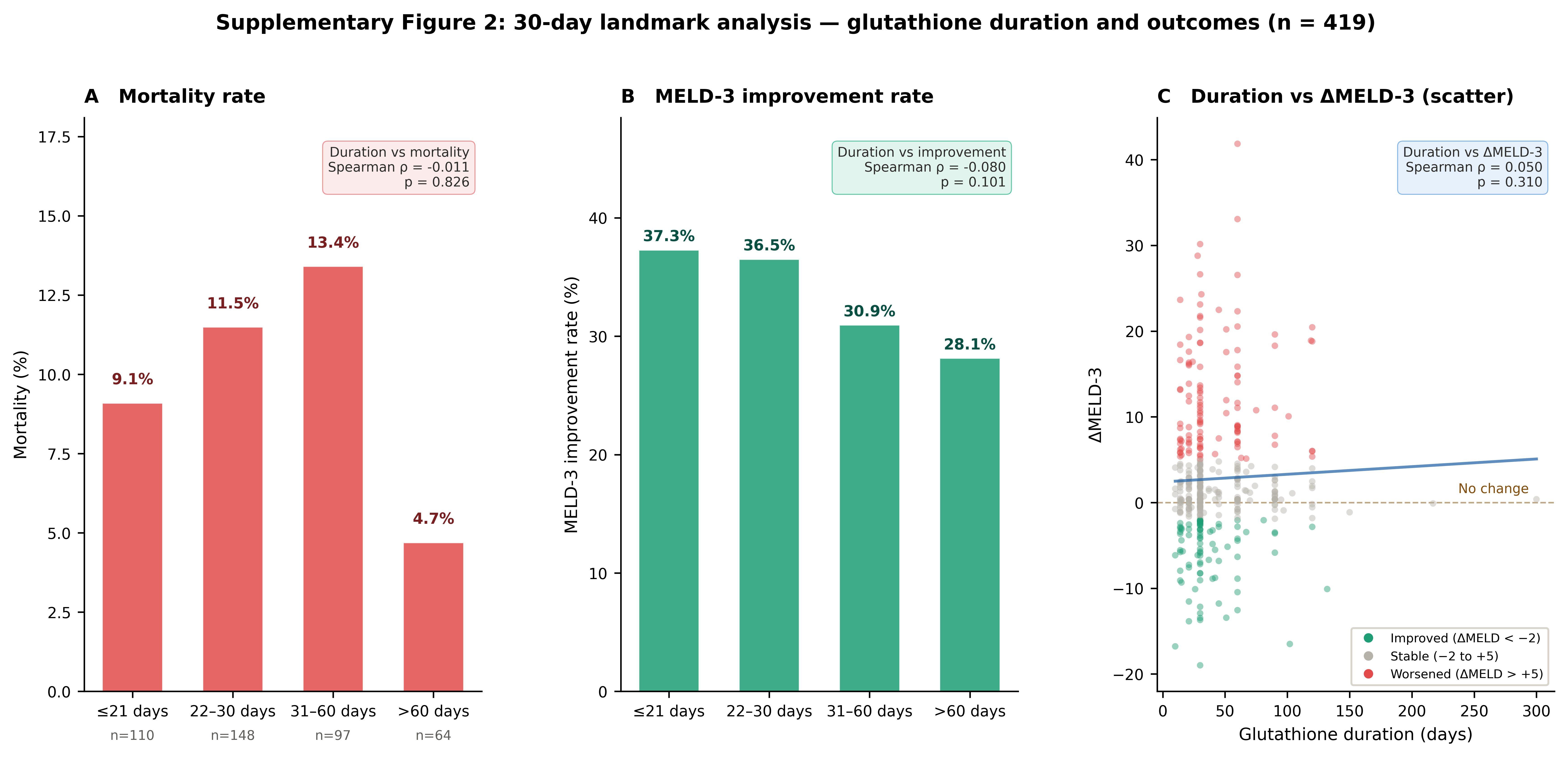

Supplement: Supplementary file 2 [file Image2.TIF]

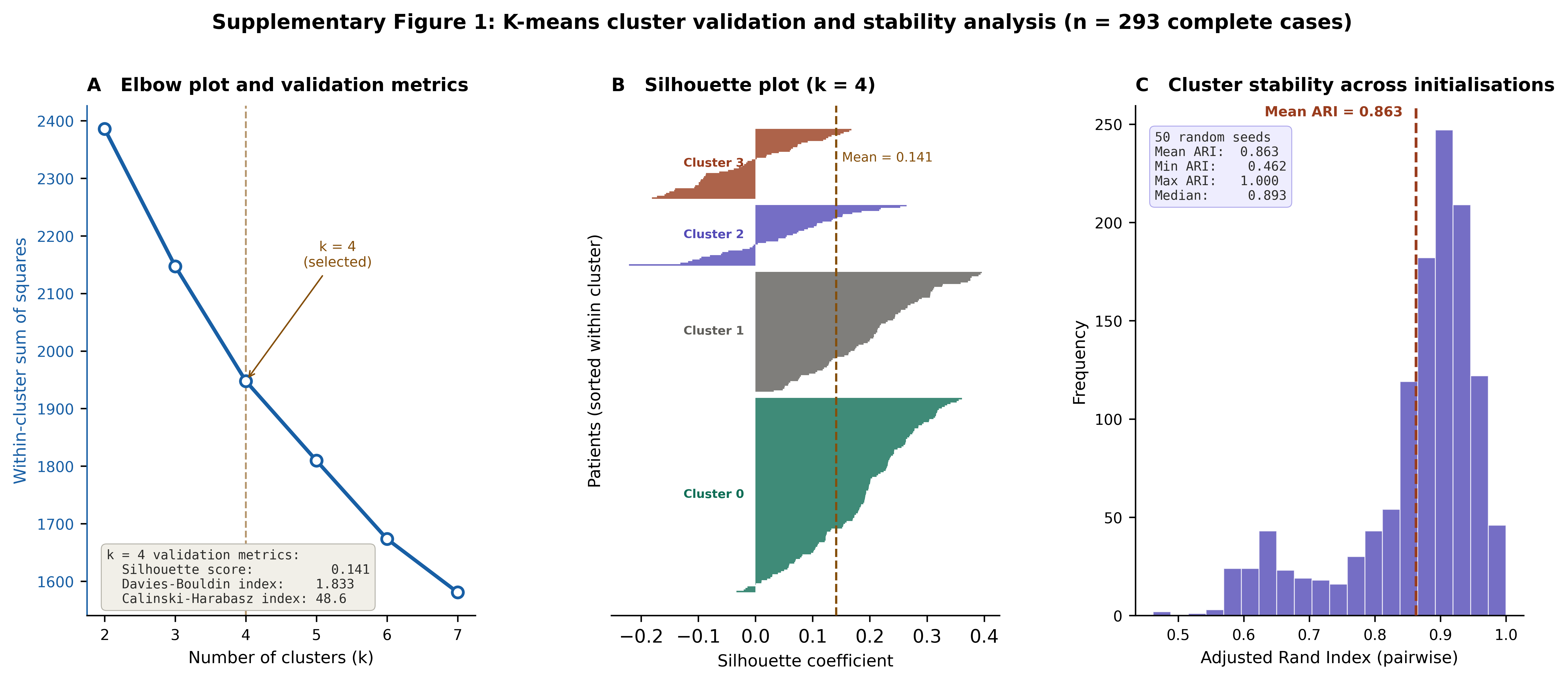

Supplement: Supplementary file 3 [file Image1.TIF]
